# Patient autonomy in self-managing their bronchial asthma exacerbation and its associated factors, 2024

**DOI:** 10.3389/falgy.2024.1483897

**Published:** 2024-10-25

**Authors:** Sadik Abdulwehab, Frezer Kedir

**Affiliations:** ^1^ School of Nursing, Institute of Health Sciences, Wollega University, Nekemte, Ethiopia; ^2^ School of Nursing, Institute of Health Sciences, Jimma University, Jimma, Ethiopia

**Keywords:** patient, autonomous, asthma, self-management, Ethiopia

## Abstract

**Introduction:**

Asthma, a global chronic respiratory condition, varies in patient autonomy due to limited resources, health literacy, and cultural beliefs, emphasizing the importance of understanding this autonomy for improved asthma management.

**Methods:**

A cross-sectional study was conducted at Jimma University Comprehensive Specialized Hospital, involving face-to-face interviews with 175 patients. Data was collected on sociodemographic characteristics, clinical factors, and autonomy levels using a validated Patient Autonomy Preference Index. Descriptive statistics and binary logistic regression analysis were used.

**Results:**

A total of 175 participants were recruited, out of them 41.7% (95% CI: 31.19, 40.41)) of participants were autonomous in managing their asthma exacerbation. 127 (72.6%) of study participants were female, with a mean age of 47.51 (SD ± 13.96), 101(57.7%) were live in urban areas, 54 (30.9%) had no formal education, 140 (80%) were married, 112 (64%) had health insurance, and 102(83.3%) obtained health information about their condition from healthcare workers. Reside in an urban area (AOR = 3.24; 95% CI: 1.40–7.49, *p* < 0.006), have health insurance (AOR = 4.30; 95% CI: 1.76–10.51, *p* < 0.001), those doing regular exercise (AOR = 4.79; 95% CI: 1.69–13.64, *p* < 0.003), have family history (AOR = 7.47; 95% CI: 1.61–34.60, *p* < 0.01), have a duration above five years since diagnosis (AOR = 0.44; 95% CI: 1.04–1.26, *p* < 0.003), and participants with a high level of health literacy (AOR = 1.10; 95% CI: 1.00–1.20, *p* < 0.042) become associated with being autonomous in managing asthma exacerbation.

**Conclusion and recommendation:**

Only around forty-two percent of study participants were autonomous in managing their asthma exacerbation. Thus healthcare providers should give due attention to those who reside in rural areas, are not insured, recently diagnosed with asthma, and with low health literacy to enhance patient autonomy and self-management practices, ultimately improving health outcomes for individuals with asthma.

## Highlights

• This study reveals that urban patients have significantly better autonomy in managing bronchial asthma compared to their rural counterparts. This finding emphasizes the impact of geographical location on health management practices, which has not been extensively highlighted in previous research.
• The research underscores the role of access to healthcare facilities and educational resources in enhancing patient autonomy.
• This study contributes valuable new insights into the dynamics of patient autonomy in asthma management while reinforcing established knowledge in the field

## Introduction

Asthma is a chronic respiratory condition causing shortness of breath and chest tightness. Environmental factors can worsen symptoms, necessitating prompt intervention (1). Autonomy in healthcare decision-making is crucial for patient-centered care and empowering patients to manage their condition independently (2).

Asthma remains a significant global health burden, affecting over 262 million people and causing nearly 455,000 deaths annually (3, 4). The management of asthma requires both clinical interventions and patient-centered approaches, emphasizing the importance of patient autonomy in managing chronic conditions like asthma which requires timely decision-making is crucial to prevent exacerbations and reduce mortality (1).

Patient autonomy in asthma management varies across healthcare settings, with high-income countries emphasizing it as a core component of chronic disease management, encouraging decision-making and independent treatment (5). In low and middle income countries, asthma management autonomy is often hindered by limited healthcare resources, low health literacy, and patient empowerment (6).

Ethiopia's asthma prevalence is between 4.6% and 9.1%, influenced by healthcare system challenges such as limited access to medications, infrastructure, and patient education, and cultural factors causing delayed interventions (7, 8). Asthma patients are at risk of severe acute attacks due to rapid air changes, necessitating prompt intervention and medication administration, and promoting patient autonomy in treatment (2, 9).

Ethiopia's healthcare system faces challenges in asthma management due to limited access to essential medications, supply chain issues, and cost (10), underdeveloped infrastructure, especially in rural areas (11), lack of patient education on asthma management can lead to delayed diagnosis and treatment and affects the level of decision-making by themselves (12).

Cultural beliefs and practices in Ethiopia may contribute to a paternalistic approach to healthcare, where patients defer to healthcare providers rather than actively participate in their care (7). In Ethiopia, there is a lack of resources for self-management of asthma, particularly during exacerbations, as many patients lack the necessary training to monitor symptoms, use inhalers correctly, and recognize medical help (13).

Misconceptions among Ethiopian asthma patients include the belief that immediate intervention is necessary for exacerbations, underestimating daily asthma management, and lacking understanding about inhalers, and cultural beliefs and stigma can further misunderstanding asthma as a transient or non-serious condition (12–14).

Poor asthma patients’ decision-making autonomy leads to exacerbations, higher healthcare utilization, reduced quality of life, poor treatment adherence, and increased healthcare costs (13, 15, 16).

National health policies may not fully emphasize patient autonomy in chronic disease management, leading to a lack of systematic efforts to promote autonomy among patients. Understanding how patients perceive their role in managing their condition and the barriers they face can inform strategies to enhance patient autonomy, ultimately leading to better asthma outcomes reduced healthcare costs, and ultimately enhancing patient outcomes and quality of life. Therefore, this study aims to assess the autonomy preferences of asthma patients and its associated factors in a tertiary hospital setting at Jimma University compressive specialized hospital in managing their condition.

## Methods and materials

### Study area and period

This research was carried out in a tertiary Hospital at Jimma University Comprehensive Specialized Hospital, Oromia, Ethiopia. Jimma city is the capital of the Jimma zone found at 356 Km from Addis Ababa, the capital city of Ethiopia, in the Southwestern part of the country. Jimma University Comprehensive Specialized Hospital is one of the oldest public hospitals in the country and it is the only teaching and referral hospital in the south-western part of the country. It provides services for approximately 19,000 inpatients, 160,000 outpatient attendances, 5,000 delivery, and 11,000 emergency cases annually. It has a bed capacity of 800 and an estimated total number of 14,000 adult patients admitted annually. The hospital has 1,600 staff members, 23 service delivery units, 698 Staff nurses, 125 pharmacy staff and different working units like medical, surgical, maternity, Gynecology, oncology, ophthalmology, psychiatry, and the like. Approximately, 19,000 patients attend the follow-up clinic for NCDs, in a year (82).

The study was conducted from May 1 to June 1 2023.

### Study design

A cross-sectional institutional research approach was employed.

### Source population

All adult patients with chronic asthma diseases at Jimma University Comprehensive Specialized Hospital.

### Study population

All selected adult patients with chronic asthma diseases were regularly followed up at Jimma University Comprehensive Specialized Hospital during the data collection period.

### Inclusion criteria

All adult patients aged ≥18 years who were regularly followed up for at least six months with chronic asthma diseases were able to respond.

### Exclusion criteria

Patients who were critically ill.

### Sample size determination

The research used a single-population proportion formula to determine the appropriate sample size, considering the level of perceived autonomy in chronic disease in Ethiopia at Mettu was 60.5% (17), with a 95% confidence interval, and a 5% margin of error. The sample size for primary potential predictors was also calculated using Epi Info Version 7.2.0.1. Subsequently, the largest sample size was selected from both methods. Later on 5% nonresponse rate was added, and the ultimate sample size was **349**. Participants were recruited over two months until the allotted number was reached.

### Sampling procedure

All adult patients aged ≥18 years with chronic asthma diseases on regular follow-ups at Jimma University Comprehensive Specialized Hospital were selected during the data collection period. The study participants were consecutively recruited from April 1 to July 1, 2023.

### Dependent Variable

Perceived level of autonomy in decision-making.

### Independent variables

Sociodemographic Variables (age, sex, monthly family income, area where they live, educational level, marital status, occupation, and source of information), clinically related variables (type and stage of the disease, family history, informal caregiver, duration of disease, type of treatment, and presence of comorbidity), health-related factors (smoking cigarettes, alcohol users, physical inactivity), patient health literacy, healthcare provider-related factors (healthcare professional role and types of healthcare profession and their role), and health information and communication (language barriers, jargon medical words, and decisional aid materials).

### Operational definitions and definitions of the terms

**Autonomy in the decision-making process refers** to a patient's ability and right to make informed, independent choices regarding their healthcare without undue influence or coercion from healthcare providers (18).
**Autonomous:** If the patients with scores above the median of the autonomy index (17).
**Not Autonomous:** If the patients with scores at or below the median (17).

### Data collection tools

The study used a Patient Autonomy Preference Index questionnaire, which has been proven to be effective in determining the level of patient autonomy in self-management. The questionnaire consists of four sections and 59 items, including socio-demographic, illness-related, health literacy, and Patient Autonomy Preference Index questionnaires (19). The Patient Autonomy Preference Index questionnaires were rated on a five-point Likert scale, with participants rating their agreement with each statement. The results were then categorized as high or low based on the mean score of each question. The instrument has improved in validity and reliability over time, making it the most effective tool for gauging patient participation in healthcare decision-making.

### Data collection techniques

Patients with brachial asthma who had been monitored for at least six months following their service completion during the data collection period were interviewed face-to-face. Two BSc nurses from Shenan Gibe Hospital were chosen based on their prior expertise in gathering data. Patient cards were used to collect clinical factor data on the patients.

#### Data quality management

The questionnaires were translated into Afaan Oromo and Amharic, and then back into English to ensure reliability. A pre-test was conducted on 10% of the sample (*α* = 0.87), including 33 participants, at Agaro General Hospital to assess data clarity, feasibility, applicability, understandability, reliability, and coherence. Data collectors received two days of training to ensure common understanding and follow the same interview procedures. The principal investigator conducted continuous follow-ups, daily reviews, and questionnaire checks to ensure accuracy and consistency throughout the data-gathering process.

#### Data processing and analyses

Before being imported into Epi Data version 4.6 and exported to SPSS version 26, the data was cleaned, verified, and coded. Descriptive and inferential statistics were used to find a linear correlation between independent variables. To test for multicollinearity, tolerance, and variance inflation factors were used. None of the variables produced a variance inflation factor >10 and tolerance <0.1 (variance inflation factor ≤2.91, and tolerance ≥0.343) were excluded. The study used binary logistic regression to determine the relationship between dependent and independent variables, and multivariable logistic regression analysis to identify factors related to patients’ autonomy in managing asthma exacerbation. The model was fit when the omnibus test was significant (*p* = 0.00), and the Hosmer-Lemeshow test was negligible (*p* = 0.406). 95% confidence intervals and odds ratios were calculated to gauge the strength of the correlation. The statistical significance threshold was established at a *p*-value less than 0.05. The findings were presented using the proper tables, charts, figures, graphs, and narrative prose.

#### Ethical consideration

This study received ethical approval from the Jimma University Research and Ethics Review Board (reference number JUIH/IRB/371/23), which also accepted the research protocol's ethics. A formal letter of collaboration was also provided, and the study was submitted to the chronic follow-up unit. Participants signed an informed consent form after being made aware of the purpose of the study. They were also told that their participation would be unaffected by their departure and that confidentiality and privacy would be upheld.

## Result

### Sociodemographic characteristics of the respondents

A total of 175 participants were recruited. Among them, 127 (72.6%) were female, the mean age was 47.51 with a standard deviation of 13.96, 92(52.6%) were Muslim followers, 101(57.7%) lived in urban areas, 54 (30.9%) had no formal education, 56(32%) of their occupation was housewife, 140 (80%) were married, 100(57.1%) had monthly income <2,500 Ethiopian Birr, 112 (64%) had health insurance, 102(83.3%) obtained health information about their condition from healthcare workers, 134(76.6%) got information from Doctors and nurses, and around 12(6.9%) not differentiate types of health care provided they receive the care and information (Table 1).

**Table 1:** Socio-demographic characteristics of adult bronchial asthma patients at the tertiary hospital of Jimma University, Ethiopia, 2023.

Variable	Category	F (%)
Sex	Male	48 (27.4)
	Female	127 (72.6)
Age( mean ± standard deviation) in years	47.51 ± 13.96	
Residence	Urban	101 (57.7)
	Rural	74 (42.3)
Occupation	Farmer	43 (24.6)
	Government employee	26 (14.9)
	Self-e1mployee	17 (9.7)
	Housewife	56 (32.0)
	Merchant	18 (10.3)
	Other(Daily labor and Student)	15 (8.5)
Educational Status	No education	54 (30.9)
	Primary	44 (25.1)
	Secondary	39 (22.3)
	Above Secondary	38 (21.7)
Marital Status	Single	22 (12.6)
	Married	140 (80.0)
	Not lived currently with their parents(Divorced and Widowed)	13 (7.4)
Monthly income(Ethiopian Birr)	Less than 2,500	100 (57.1)
	Greater than and equal to2,500	75 (42.8)
Health Insurance	Yes	112 (64.0)
	No	63 (36.0)
Source of information about the disease on follow-up	Health Care worker	102 (58.3)
	social media and Health Care worker	75 (41.7)
Types of health care provider they got information about their disease	They differentiate types of their Health Care Providers (whether Nurse/Doctor)	163 (93.1)
	They don’t differentiate types of their Health Care Provider	12(6.9)

### Risk health behavior of the adult with bronchial asthma

Regarding risky health behavior, 172(98.3%) were non-cigarette smokers, 18(10.3%) were non-alcohol users, from those who used alcohol 23 (8.6%) drank alcohol for more than five years, 39(22.3%) engaged in regular physical exercise and from those 37(21.1%) was doing physical exercise less than 30 min, and 99(56.6%) not done regular exercise due to not have interest with doing exercise (Table 2).

**Table 2:** Risk health behavior of adult bronchial asthma at the tertiary hospital of Jimma University, Ethiopia, 2023.

Risky health behavior	Category response	Frequency (%)
Smoking status	Yes	3 (1.7)
	No	172 (98.3)
Duration of smoking (years)	1 Up To 5 Years	1 (0.6)
	Above 5 Years	2 (1.1)
Current smokers	Yes	1 (0.6)
	No	2 (1.1)
Alcohol drinking status	Yes	18 (10.3)
	No	157 (89.7)
Duration of alcohol (years)	Less Than 5 Year	3 (13.1)
	Greater Than And Equal To 5 Year	23 (8.6)
Current drinker	Yes	14 (8)
	No	12 (6.9)
Regular exercise	Yes	39 (22.3)
	No	136 (77.7)
Duration Regular Exercise (years)	Less Than 30 Minutes	37 (21.1%)
	Greater Than 30 Minutes	7 (4%)
Reason for not doing exercises	Illness	27 (15.4%)
	Not Interested	99 (56.6%)
	Not Appropriate Place	5(2.9%)

### Clinical related factors of the participant of adult with bronchial asthma

From the total recruited participants 72(41.1%) had comorbid disease with asthma, 80(45.7%) had health information material for decision for disease on follow-up and 20(11.4%) for comorbid disease with asthma, 17(9.7%) had family history of the same illness, 43(24.6%) of their caregiver was family, 41(23.4%) has peer network with the same disease on follow-up, 108 (61.7%) diagnosed below five years after diagnosis, and 146 (83.4%) had a history of hospitalization for the disease on follow-up (Table 3).

**Table 3:** Clinical features of participants with bronchial asthma disease at the tertiary hospital of Jimma University, Ethiopia, 2023.

Clinical characteristics	Response	Frequency (%)
Comorbidity other than disease on follow-up	Yes	72 (41.1)
	No	103 (58.9)
Having health information material for decision for disease on follow-up	Yes	80 (45.7)
	No	95 (54.3)
Having health information material for decision for comorbid disease	Yes	20 (11.4)
	No	53 (30.3)
Family history of the same illness	Yes	17 (9.7)
	No	157 (89.7)
Informal caregivers	Family	43 (24.6)
	Not family members	131 (74.9)
Peer network with the same disease on follow-up	Yes	41 (23.4)
	No	134 (76.6)
Duration(year)	<5 years	65 (38.3)
	≥5 years	108 (61.7)
History of hospitalization with the disease on the follow-up	Yes	146 (83.4)
	No	29(16.6)

### Level of patient health literacy

Items related to health literacy, which were rated from one to five on a five-point Likert scale, were totaled and handled as continuous variables. The study participants’ health literacy mean and standard deviation was 20.923 (SD ± 5.31).

### Patient preference for autonomy

The participants’ preference for autonomy in managing their asthma exacerbation was found to have a mean score and standard deviation of 43.87 (SD ± 10.49) with a range of 23–72. By dichotomizing based on their mean, 73(41.7%) [95% CI: 34.34, 49.09] Of study participants reported having autonomy in their self-management of asthma exacerbation.

### Factors associated with patient autonomy in managing their asthma exacerbation

By adjusting for confounding factors, binary and multivariable logistic regression models were used to evaluate the relationship between independent variables and patient autonomy in self-management. Age, residence, educational status, marital status, occupation, health insurance, source of health information, types of health care provider in information sharing, alcohol, exercise, comorbidity, having decisional aid for disease on follow-up, Family history with the disease on follow up, peer network, duration since diagnosed, hospitalization, and patient health literacy were a candidate for multivariate logistic regression analysis. In the multivariate logistic regression analysis, only six variables (residence, health insurance, regular exercise, family history, duration of the disease since diagnosis, and patient health literacy became statistically significant (*p* < 0.05) (Table 4).

**Table 4:** Multivariable analysis of variables statistically significantly associated with asthma patient autonomy in their self-management at the tertiary hospital of Jimma University, Ethiopia, 2023.

Variable	Categories	Patient autonomous in self-managing brachial asthma		OR with 95% with 95% CI		*p* value
		Not autonomous (%)	Autonomous (%)	COR (95%CI)	AOR (95%CI	
Residence	Urban	43 (42.6%)	58 (57.4%)	5.31 (2.66–10.58)	3.24 (1.40–7.49)	0.006
	Rural	59 (79.7%)	15 (20.3%)	1	1	
Health insurance	Yes	57 (50.9%)	55 (49.1%)	2.41 (1.25–4.67)	4.30 (1.76–10.51)	0.001
	No	45 (71.4%)	18 (28.6%)	1	1	
Regular Exercise	Yes	12 (30.8%)	27 (69.2%)	0.23 (0.11–0.49)	4.79 (1.69–13.64)	0.003
	No	90 (66.2%)	46 (33.8%)	1	1	
Family History	Yes	3 (17.6%)	14 (82.4%)	7.97 (2.20–28.90)	7.47 (1.61–34.60)	0.010
	No	99 (63.1%)	58 (36.9%)	1	1	
Duration	<5 year	50 (74.6%)	17 (25.4%)	0.57 (0.23–1.57)	0.44 (1.04–1.26)	
	≥5 year	52 (48.1%)	56 (51.9%)	1	1	0.003
Health literacy	Mean ± SD = 21.97 ± 6.5448			1.20 (1.11–1.29)	1.10 (1.00–1.20)* *β* = 0.093	0.042

N. B= * shows the statistically significant variables, with *p*-values less than 0.005 indicating statistical significance. Health literacy levels were treated as continuous variables. AOR, Adjusted Odd Ratio; CI, Confidence Interval; COR, Crude Odd Ratio; OR, Odd Ratio; SD, Standard Deviation.

Participants who live in the urban area has three times more likely autonomous in self-management of asthma exacerbation than those from rural areas (AOR = 3.24; 95% CI: 1.40–7.49), those participants had health insurance has four times more likely autonomous in self-management of asthma exacerbation than those has no health insurance (AOR = 4.30; 95% CI: 1.76–10.51), those doing regular physical exercise has around four point seven more likely autonomous in self-management of asthma exacerbation than those not doing regular physical exercise (AOR = 4.79; 95% CI: 1.69–13.64).

Participants those has family history of bronchial asthma seven ctimes more likely autonomous in self-management of asthma exacerbation than those has no family history of asthma (AOR = 7.47; 95% CI: 1.61–34.60), those participants diagnosed with bronchial asthma below five year duration was 56% less likely autonomous in self-management of asthma exacerbation than those five years and above since diagnosed with asthma (AOR = 0.44; 95% CI: 1.04–1.26).

This study also indicated, for every one-point increase in a participant's health literacy score, the Log odds that the participants will have autonomous in self-management of asthma exacerbation increases by 1.1(*β* = 0.093, AOR = 1.1, 95% CI: 1.00–1.20) on average (Table 4).

## Discussion

This research was done at the Jimma Comprehensive Specialized Hospital in Jimma, Oromia, Ethiopia, to evaluate patient autonomy in self-management of asthma exacerbation and the related variables among patients with asthma. According to the study's results, 73 participants, or 41.7%, were autonomous in self-management of their disease. This finding is relatively similar to other studies conducted in Turkey reported that only 35.6% of asthma patients demonstrated autonomy in self-management decisions (20), in Malaysia found that 39.3% of asthma patients exhibited autonomy in managing their condition (21), and Mettu Karl Hospital in Ethiopia reported that only 29.5% of asthma patients were autonomous in self-management (17). This due to educational, cultural, socioeconomic, and healthcare system-related factors, limiting informed health decisions. So it needs to enhance asthma management knowledge, train healthcare providers, reduce socioeconomic barriers, provide affordable medications, strengthen primary healthcare, incorporate psychosocial support, and prioritize patient autonomy in chronic disease management.

The findings of this study showed that participants from urban areas were more likely to be reported as having autonomy in self-management of asthma exacerbation than those from rural areas, similar to findings in previous studies in Ethiopia (22), Nigeria (23), and Malawi (24). The study highlighted that urban patients had better access to healthcare facilities, educational resources, and information, which contributed to better self-management practices and decision-making autonomy. Thus healthcare providers should increase the availability and accessibility of healthcare services in rural areas by enhancing infrastructure, establishing more clinics, and providing mobile health units.

According to the study's findings, participants with health insurance were more likely to be autonomous in managing their asthma attacks than those without insurance. These findings are consistent with research done in Nigeria (25), the UK (26), and Chicago (27). The reasons were that insured patients often benefit from better access to medications, regular medical follow-ups, and health education programs, allowing more frequent interactions with healthcare providers, which enhances patient education and self-management skills. By expanding insurance coverage and improving access to essential healthcare resources, patient autonomy in asthma management can be significantly enhanced.

The study found a correlation between the status of regular exercise and patients’ autonomy in self-managing asthma, which is consistent with findings from studies conducted in the United States (28), Canada (29), and Finland (30). These studies indicated that regular exercise enhances patients’ confidence and ability to manage asthma independently due to improved lung function and physical fitness. Healthcare providers incorporate tailored exercise routines into asthma management plans. These programs should be designed to improve lung function and physical fitness, thereby boosting patients’ confidence and autonomy in managing their condition. Educating patients on safe and appropriate exercises, along with regular follow-ups, can further reinforce self-management skills.

The study's findings indicate that individuals with a family history of asthma exhibit greater autonomy in managing asthma attacks compared to those without such a history. This aligns with several studies that emphasize the role of family dynamics in asthma management as studies done in the UK (31), Ecuador (32), and Pakistan (33). These findings collectively underscore the importance of familial influence in asthma management, suggesting that family history may foster better self-management practices. This encourages healthcare providers to actively engage families in asthma education and management plans, especially for patients with a family history of the condition. Involving family members in care routines, decision-making, and educational sessions can enhance patient autonomy by fostering a supportive environment that reinforces effective self-management practices.

The results of this study demonstrated that individuals who were diagnosed with asthma more than five years ago are more autonomous in managing their asthma attacks than their counterparts, which is similar to the findings of studies conducted in Ecuador (32), Indonesia (34, 35), UK (36). This is due to individuals with a longer duration of asthma typically having more experience managing their condition, they develop and refine self-management skills, often learn to adapt their treatment plans based on their evolving needs and develop greater self-efficacy or confidence in their ability to manage their health. Offer continuous self-management training and education programs for patients at various stages of their asthma journey, empowering them with the necessary experience and confidence for autonomous disease management.

According to the study's findings, asthmatic patients become more autonomous in managing asthma attacks as their health literacy level rises. These results are consistent with those found in Australia (37), UK (38), Nepal (39), and Germany (40) highlighting that improved health literacy significantly influences decision-making in accessing emergency healthcare for asthma exacerbations, emphasizing the role of self-management strategies developed through past experiences and education. This collectively shows the importance of health literacy in empowering asthmatic patients to manage their condition effectively. Implement health literacy programs to educate patients on asthma management, treatment options, and emergency response strategies, empowering them to make informed decisions and manage asthma independently, tailored to different literacy levels.

### Strengths and limitations of the study

The study used a cross-sectional institutional research design to examine patient autonomy in self-management, specifically in asthma patients. The research was conducted in a tertiary hospital, allowing for a diverse patient population. The Patient Autonomy Preference Index was used to understand decision-making and self-management preferences. The study included patients with at least six months of regular follow-up, ensuring reliability and exploring the relationship between autonomy and asthma management. However the exclusion of critically ill patients may limit the applicability of the findings to a broader patient population, the cross-sectional design does not allow for causal inferences, as it captures data at a single point in time, and Potential biases in self-reported data could affect the accuracy of the results, as patients may overestimate their autonomy.

The study's limitation is the use of the Patient Autonomy Preference Index, which may not be specific to bronchial asthma management. The tool does not account for unique aspects of asthma care, such as adherence to individualized action plans or specific guidelines. The study also did not evaluate adherence behaviors, such as medication use or proper inhaler technique. Future research should adapt Patient Autonomy Preference Index to better reflect asthma care and recruit a larger cohort to further validate these findings and enhance their generalizability.

### Implications for nursing practice

Nurses should be aware of the factors influencing patient autonomy, such as health literacy and access to healthcare, to better support patients in self-management and also Training programs focusing on enhancing communication skills can empower nurses to facilitate shared decision-making with patients, particularly in rural settings where barriers exist.

#### Conclusion and recommendation

Only around forty-two percent of study participants were autonomous in managing their asthma exacerbation. Thus healthcare providers should give due attention to those who reside in rural areas, are not insured, recently diagnosed with asthma, and with low health literacy to enhance patient autonomy and self-management practices, ultimately improving health outcomes for individuals with asthma.

## Data Availability

The raw data supporting the conclusions of this article will be made available by the authors, without undue reservation.
